# Extracellular Matrix With Bone Marrow Aspirate Concentrate for Osteochondritis Dissecans of the Capitellum in Adolescents

**DOI:** 10.1002/atn2.70103

**Published:** 2026-06-28

**Authors:** Joshua T. Bram, Nicolas Pascual‐Leone, Nathan H. Varady, Colson Zucker, Peter D. Fabricant

**Affiliations:** ^1^ Lerner Children's Pavilion Hospital for Special Surgery New York New York U.S.A.

## Abstract

Capitellar osteochondritis dissecans is most frequently encountered in overhead and tumbling adolescent athletes. Traditional management with abrasion chondroplasty or microfracture results in fibrocartilage formation and variable return to sport rates, whereas osteochondral autograft transfer system or osteochondral allograft transplantation may incur unnecessary morbidity for small, shallow lesions. This article therefore describes the application of a biologic scaffold composed of extracellular matrix combined with bone marrow aspirate concentrate to unstable, shallow (<6‐7 mm), but contained capitellar osteochondritis dissecans lesions through an anconeus‐splitting arthrotomy. This technique provides excellent exposure of the capitellum and offers the potential for biologically favorable hyaline‐like cartilage regeneration. With promising early clinical results, this approach is more easily implemented even for those without extensive elbow arthroscopy experience and represents a favorable middle‐ground between microfracture and osteochondral autograft transfer system/ osteochondral allograft for adolescent athletes with capitellar osteochondritis dissecans lesions.

## Graphical abstract

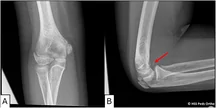

**VIDEO 1:** Technique for extracellular matrix with bone marrow aspirate concentrate reconstruction of capitellar osteochondritis dissecans lesions of the right elbow in an 11‐year‐old male right hand dominant baseball pitcher. Video content can be viewed at https://doi.org/10.1002/atn2.70103.

Osteochondritis dissecans (OCD) of the humeral capitellum is frequently encountered among baseball‐playing males and older adolescents.^1^ Though microfracture has long been the gold‐standard treatment, this relies on the formation of fibrocartilage over more anatomic hyaline cartilage, and clinical outcomes have been mixed likely due to abnormal host bone in the setting of OCD.^2^ Additionally, for shallower, contained lesions without deeper bony involvement, osteochondral autograft transfer system (OATS) or osteochondral allograft (OCA) procedures are likely excessive and are associated with donor site morbidity with autograft harvest and radius of curvature mismatch. Recent literature has revealed extracellular matrix (ECM) with bone marrow aspirate concentrate (BMAC) to be a viable treatment option for capitellar OCD lesions, though few detailed surgical descriptions exist.^3^ This article therefore reviews the technique for the use of ECM‐BMAC for contained, unstable capitellar OCD lesions in adolescent patients.

## SURGICAL TECHNIQUE

### Indications

**FIGURE 1:**
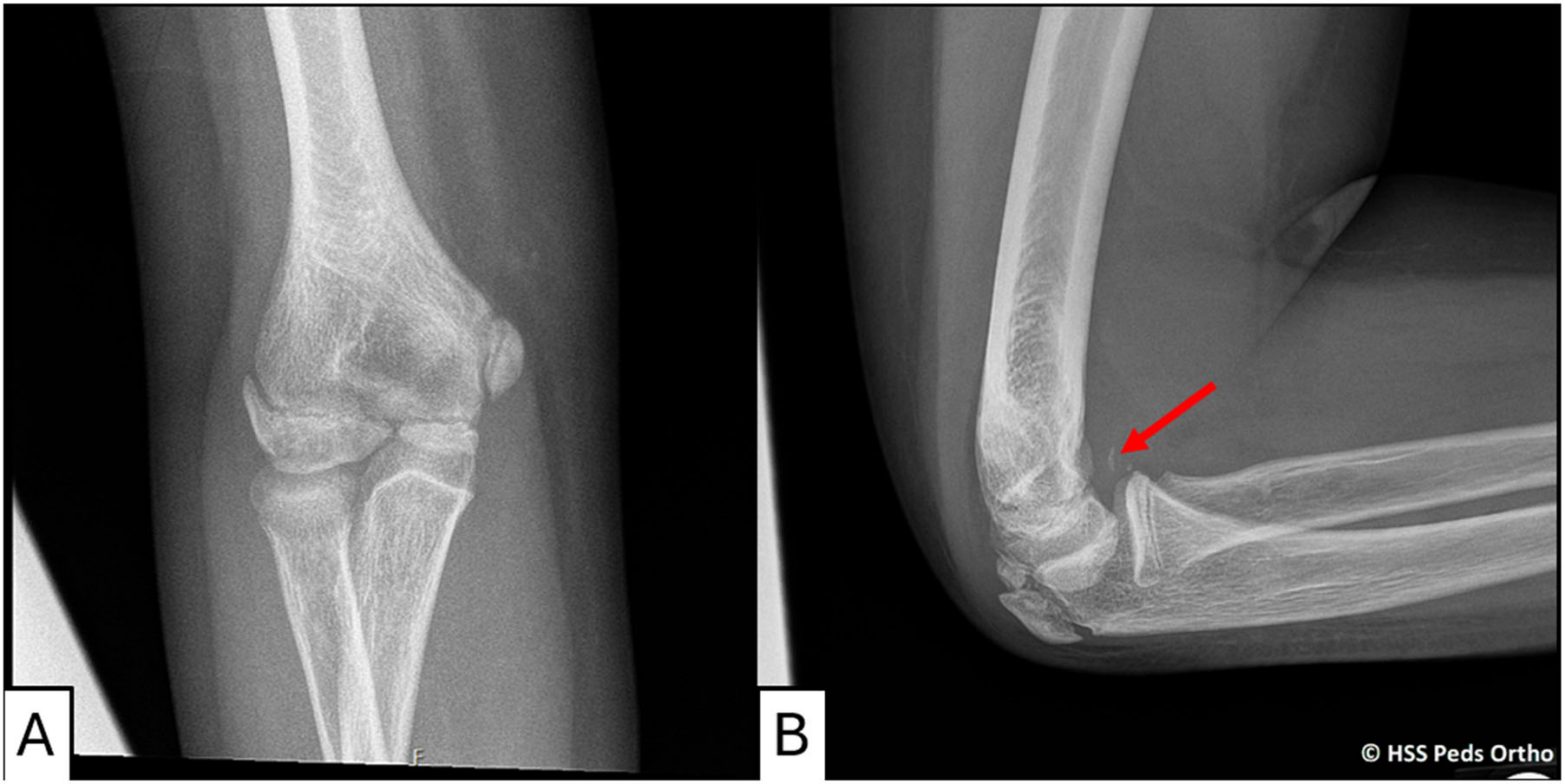
X‐rays ((A) anteroposterior, (B) lateral) of an 11‐year‐old right‐hand‐dominant male baseball pitcher with an unstable capitellar OCD lesion of the right elbow and evidence of an anterior loose body (B, arrow). (OCD, osteochondritis dissecans.)

**FIGURE 2:**
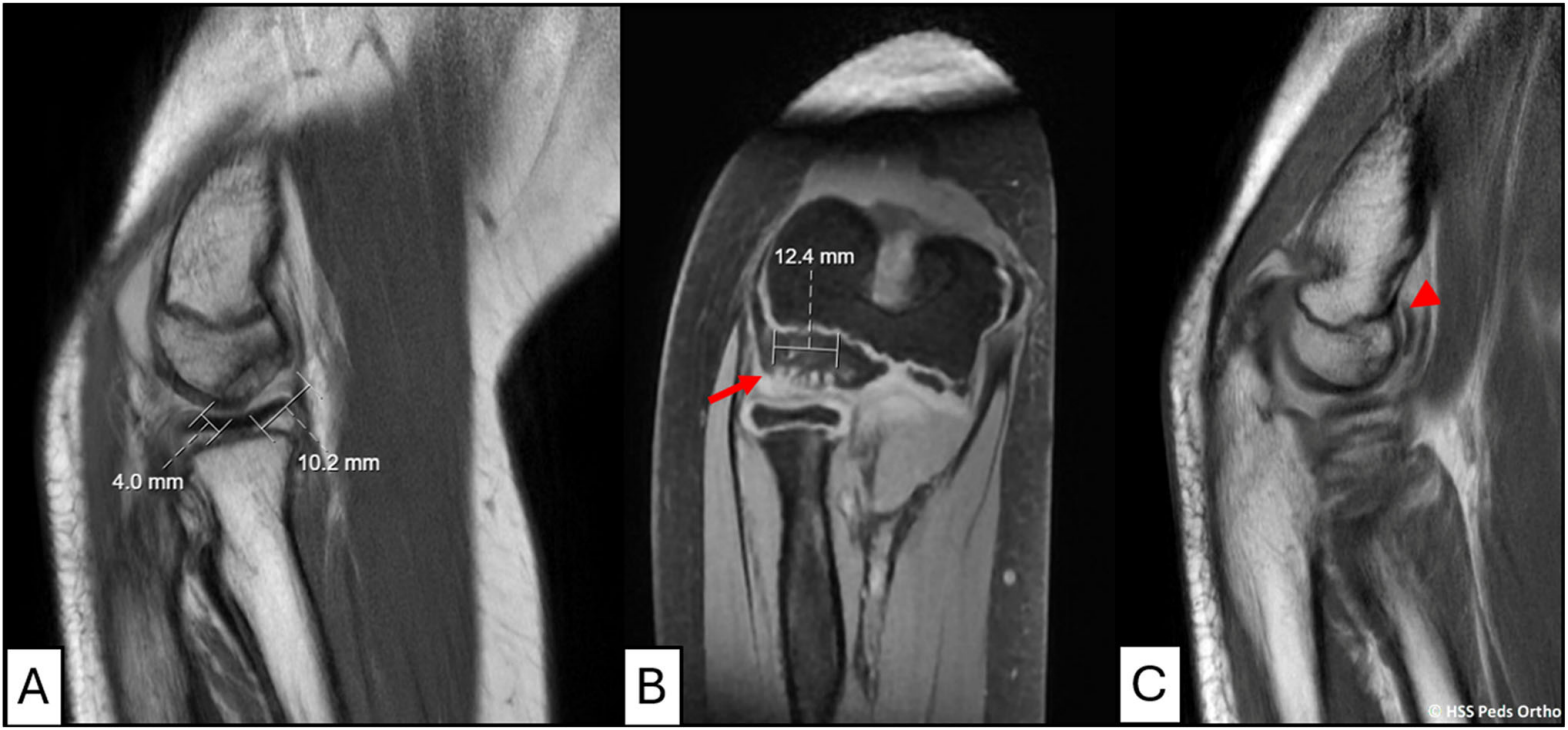
Sagittal proton density MRI sequence (A) displays a 4 mm deep, 10 mm anterior to posterior OCD lesion of the capitellum extending to the 3 o'clock position proximally. A fast spoiled gradient echo fat‐saturated coronal sequence (B) shows the lesion to be contained with an intact radial border (arrow), measuring approximately 12 mm medial to lateral. This sagittal PD sequence (C) shows an approximately 1 × 1 cm loose body in the anterior compartment (arrowhead). (MRI, magnetic resonance imaging; OCD, osteochondritis dissecans; PD, proton density.)

Patients with stable‐appearing capitellar OCD lesions on magnetic resonance imaging (MRI) without mechanical symptoms or an intra‐articular loose body are initially managed conservatively with sports discontinuation/avoidance of overhead activities. Physical therapy is initiated, and a repeat MRI is obtained in 3 months to assess lesion healing. For unstable lesions with displaced loose bodies (or loose bodies in situ), operative intervention is recommended (Figures 1 and 2). For smaller, shallow, contained lesions surrounded circumferentially by healthy cartilage, the senior author favors treatment with ECM‐BMAC. For uncontained lesions or those that are unsalvageable (Hefti grades 3‐4) with deeper subchondral bone involvement, an OATS procedure is typically performed.

### Positioning and Setup

**FIGURE 3:**
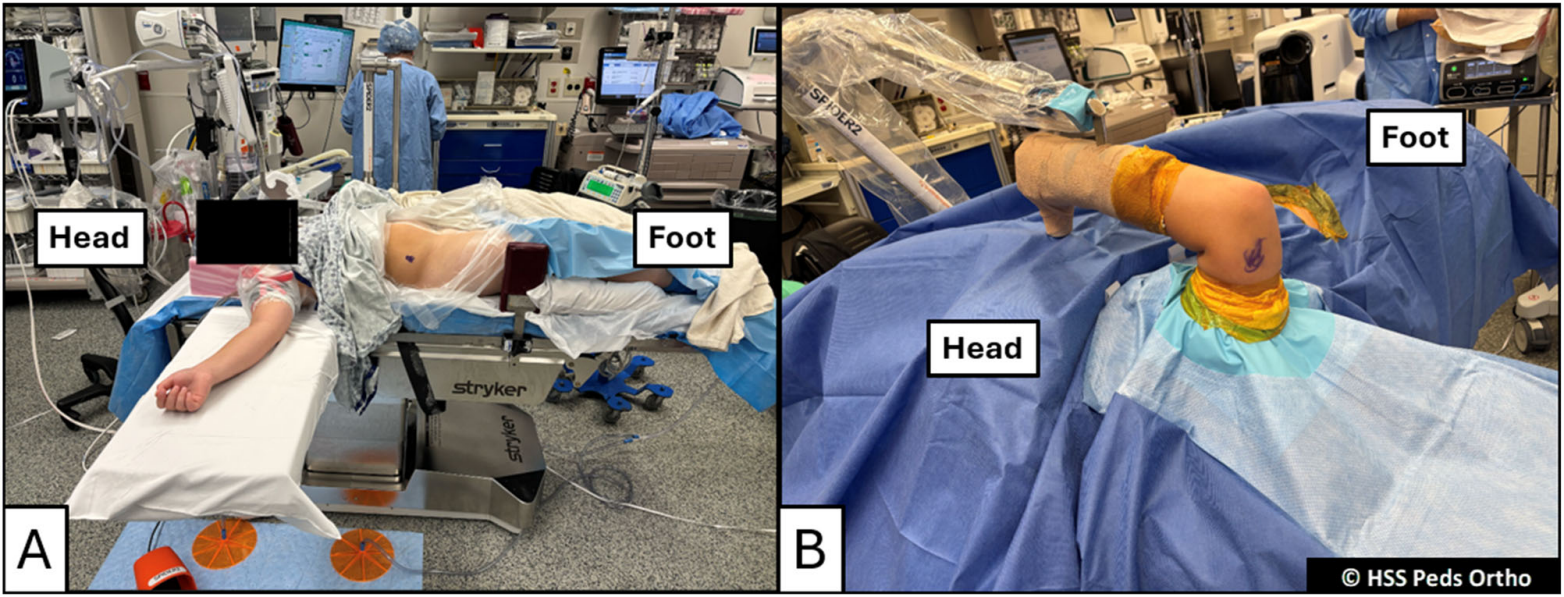
The patient is positioned supine on a standard operating room table with hand table extension and turned 90° such that the operative extremity (right arm for this patient) extends into the room (A). The arm is then secured in a pneumatic elbow positioner fashioned to the opposite side of the table and the patient's arm flexed to 90° (B).

At our institution, anesthesia performs an infraclavicular nerve block in addition to the induction of general anesthesia. The patient is positioned supine on a standard operating room table turned 90° such that the operative extremity extends into the room (Figure 3). Both the arm and iliac crest are sterilely prepped and draped. The arm is then secured in a Spider elbow positioner (Smith & Nephew, Watford, UK) fashioned to the opposite side of the table and the patient's arm flexed.

### Iliac Crest Bone Marrow Aspiration

A Jamshidi‐style needle (Arthrex Angel cPRP and Bone Marrow Processing System, Arthrex, Naples, FL) is introduced into the superior iliac crest 2 to 3 cm posterior to the anterior superior iliac spine. Approximately 60 mL of bone marrow aspirate is then withdrawn and concentrated into BMAC using the Arthrex bone marrow processing system on the back table. Hemostasis is obtained at the aspiration site, and sterile dressings are applied.

### Elbow Arthroscopy

**FIGURE 4:**
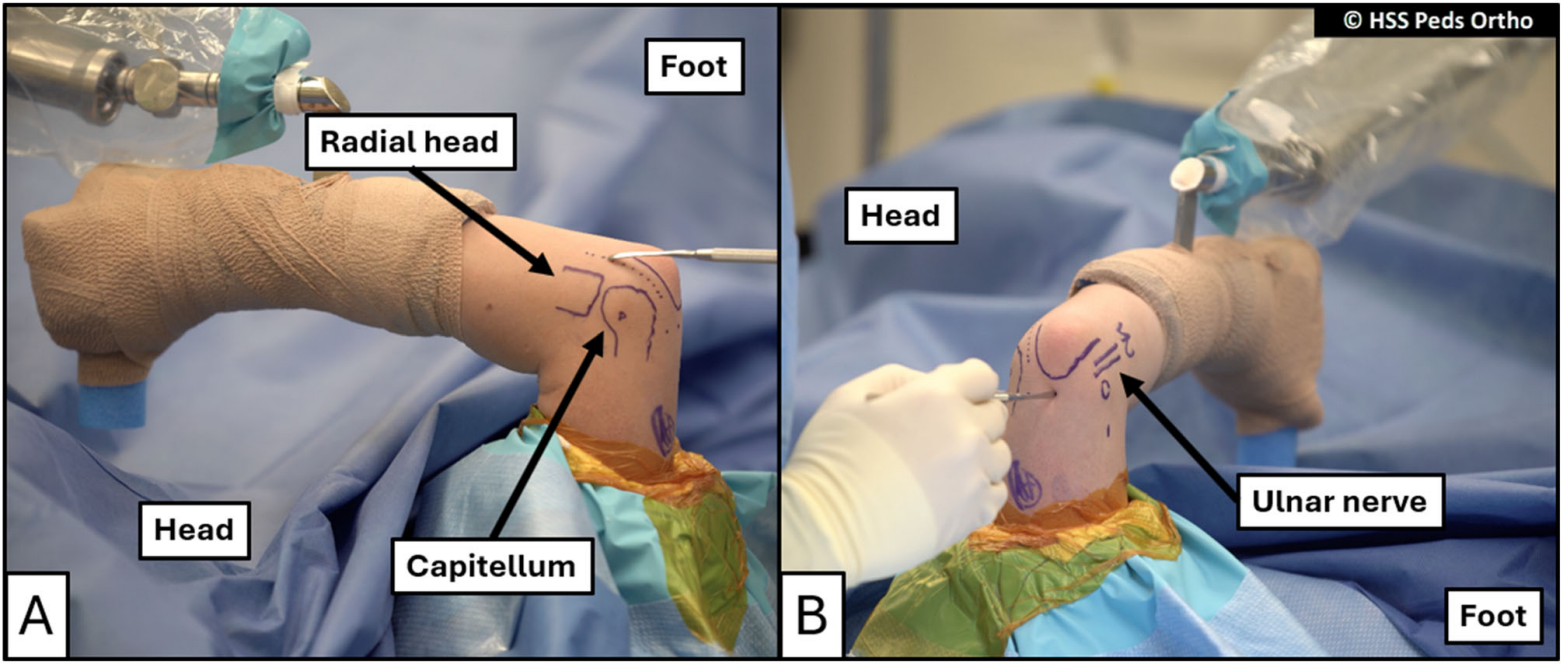
Relevant landmarks are marked. The lateral epicondyle, capitellum, and radial head are identified (A), and the planned incision for the elbow arthrotomy is marked, located halfway between the lateral aspect of the radiocapitellar joint and the posterior border of the ulna. The medial epicondyle is also identified (B), in addition to the ulnar nerve and the location of the proximal anteromedial portal (2 cm proximal and 1 cm anterior to the medial epicondyle).

**FIGURE 5:**
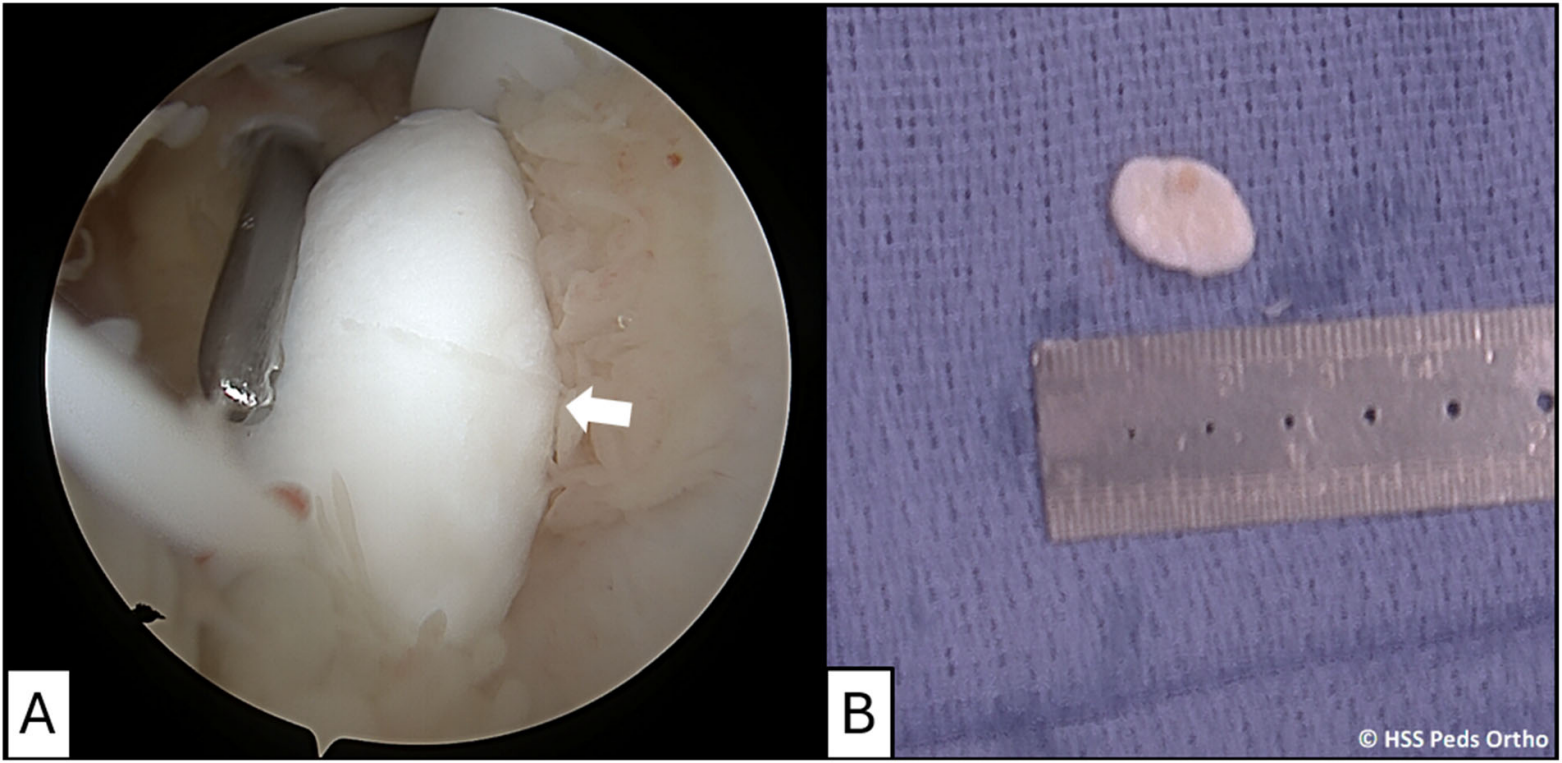
During elbow arthroscopy, the ulnohumeral and radiocapitellar joints (anterior compartment) are evaluated for the presence of loose bodies (A). In this patient, an approximately 1 cm loose body (white arrow) was identified and removed with a straight snap (B).

Every case begins with a diagnostic elbow arthroscopy (Video 1). Approximately 20 mL of sterile saline is injected to distend the elbow joint via the planned posterior portal through the triceps tendon. A standard anteromedial portal is then created 1 cm anterior and 2 cm proximal to the medial epicondyle, anterior to the intermuscular septum to avoid iatrogenic injury to the ulnar nerve (Figure 4). We favor the use of a 4 mm, 30° arthroscope for visualization. An anterolateral portal is then created under direct visualization 1.5 cm proximal to the lateral epicondyle and anterior to the humerus. The ulnohumeral and radiocapitellar joints (anterior compartment) are then evaluated for the presence of loose bodies, synovitis, and other cartilage injury potentially requiring debridement (Figure 5). Attention is then turned to the posterior compartment through the posterior portal through the triceps tendon, which can harbor additional loose bodies. Care must be taken to examine the MRI preoperatively for the presence of a perforate or nonossified olecranon fossa to avoid iatrogenic injury to the anterior neurovascular structures when making this portal. After this is completed, the remainder of the procedure is done via a mini‐open anconeus‐split arthrotomy.

### Elbow Arthrotomy

**FIGURE 6:**
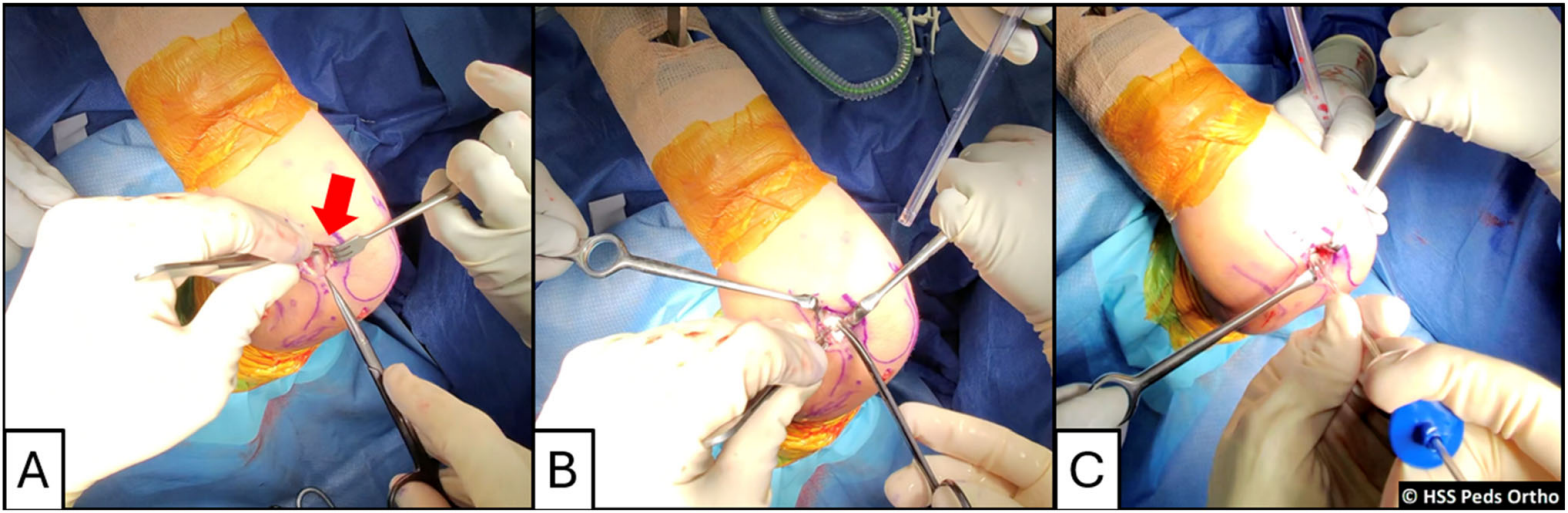
After the 2 to 3 cm skin incision is made, the anconeus fascia (red arrow) is divided followed by splitting the anconeus muscle belly in line with its fibers (A). A capsulotomy is then made to enable visualization of the affected capitellum (B). After adequate preparation and removal of devitalized tissue, the previously prepared ECM‐BMAC is applied into the defect (C). (ECM‐BMAC, extracellular matrix with bone marrow aspirate concentrate.)

A 2‐ to 3‐cm posterolateral incision is made overlying the radiocapitellar joint. An anconeus‐split approach is preferred (Figure 6). After dissection through the dermis, the anconeus fascia is divided with a scalpel. The anconeus muscle is then split in line with its fibers, and a capsulotomy is made. Using the arm positioner, the elbow can then be progressively flexed to allow complete visualization of the OCD lesion.

### ECM With BMAC

**FIGURE 7:**
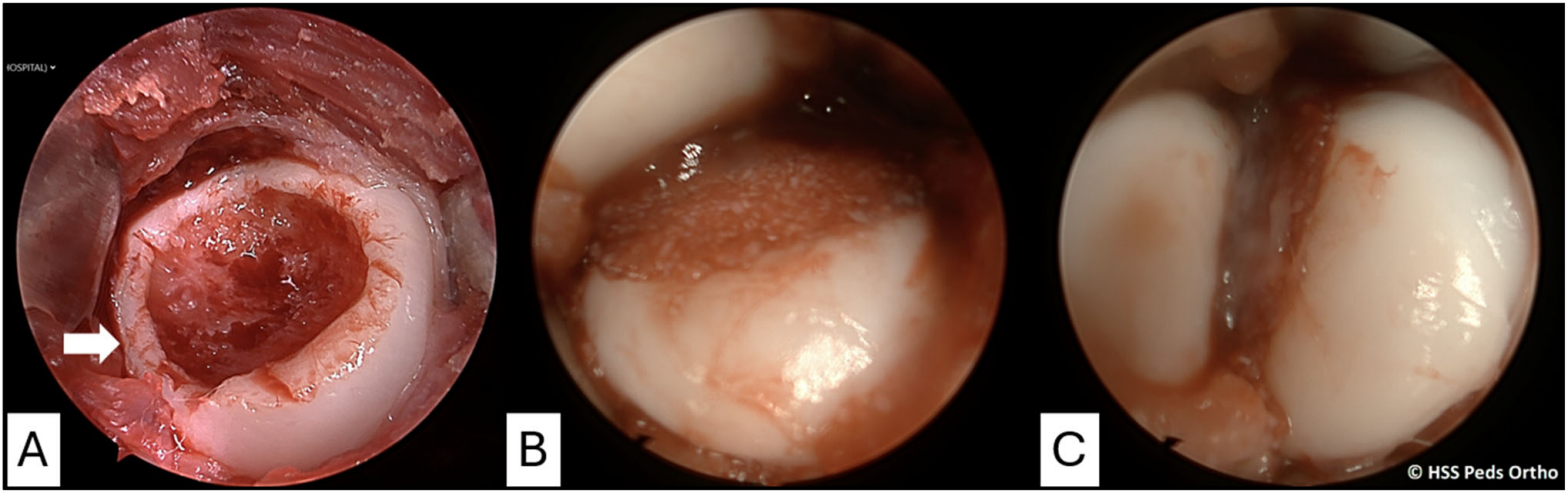
These images illustrate a contained capitellar OCD lesion affecting the right elbow of an 11‐year‐old male obtained after performing the anconeus split approach and arthrotomy. The lesion bed is prepared with a combination of curettes, scalpel, and burr as necessary down to a stable base of bleeding subchondral bone (A) with stable vertical cartilage edges (intact radial border noted with white arrow). The ECM‐BMAC mixture is applied and smoothed with a freer elevator to a flush or slightly recessed position and sealed with Fibrin glue (B,C). (ECM‐BMAC, extracellular matrix with bone marrow aspirate concentrate.)

Curettes are used to debride the lesion down to a stable base of bleeding subchondral bone with stable vertical cartilage edges (Figure 7). After adequate preparation and removal of devitalized tissue, the lesion is irrigated and dried. The previously harvested BMAC is then mixed with BioCartilage ECM (Arthrex, Naples, FL) and mixed to the consistency of a putty. This is then applied into the defect, to be flush or slightly recessed to the surrounding cartilage. Tisseel fibrin glue (Baxter International, Deerfield, IL) is placed over the lesion and allowed to cure.

### Closure and Postoperative Protocol

The arthrotomy is closed using interrupted 2‐0 absorbable braided sutures, and the anconeus fascia is closed using a similar running suture. Layered closure is then completed with 3‐0 absorbable braided sutures for the dermal layer and 3‐0 absorbable monofilament suture for the subcuticular layer. All portals are closed using a 3‐0 absorbable monofilament suture. The patient is then placed in a posterior slab plaster splint at 90° of flexion and neutral forearm rotation for 10‐14 days and instructed to remain nonweight‐bearing, at which time they are seen for splint removal and clinical examination. After 2 weeks, physical therapy is initiated emphasizing gentle range of motion until 6 weeks postoperatively. From 6 to 12 weeks, patients may begin nonimpact strengthening exercises and gradual resumption of weight‐bearing as tolerated. Clearance to return to sport is typically achieved at 5 to 6 months postoperatively.

## DISCUSSION

This article provides a technique guide for the treatment of shallow (<6‐7 mm in depth), unstable but contained capitellar OCD lesions with ECM and BMAC through an anconeus‐splitting approach.^3^ This technique provides excellent visualization and allows for hyaline‐like cartilage generation at the lesion site.

Management of capitellar OCD lesions is overall heterogenous. More historical treatment with arthroscopic debridement with or without isolated microfracture is associated with variable outcomes and return to sport rates.^3^ At a mean 11.5‐year follow‐up, Matsuura et al. reported on the outcomes of arthroscopic debridement of capitellar OCD in adolescent baseball players, observing that although 87% of patients returned to competitive baseball, only 1 of 5 pitchers was able to return.^4^

Conversely, the treatment of capitellar OCD lesions with OATS or OCA has shown more favorable outcomes. Bae et al. observed 93% complete graft incorporation on postoperative MRI and no complications for 28 adolescent patients treated with OATS.^5^ Although fewer studies have reported on the performance of OCA, Mirzayan et al. described its application in 35 cases with 100% graft incorporation, 100% return to sport at the same level or higher, and improved patient‐reported outcomes.^6^ It is our practice to perform OATS/OCA for uncontained lesions or those with deeper, bony involvement and cyst formation.

For shallow and contained lesions, few studies have reported outcomes of ECM and BMAC grafting for capitellar OCD lesions.^7^ Caldwell et al. described the all‐arthroscopic use of micronized allogeneic cartilage scaffold for treatment of capitellar OCD lesions^8^ but did not report on outcomes or an open approach that can be more broadly applied to those without extensive elbow arthroscopy experience. Our group previously revealed that treatment of capitellar OCD lesions with this ECM and BMAC approach led to significantly improved 1‐year postoperative range of motion and 90% return to sport rate at a mean of 5.8 months among 20 adolescents.^1^ Such findings show the effectiveness of this technique, though additional studies with long‐term outcomes are still necessary. Advantages and limitations of the technique are found in Table 1.

**TABLE 1:** Advantages, Limitations, Pearls, and Pitfalls

Advantages
‐Promotes hyaline‐like cartilage regeneration compared to fibrocartilage with microfracture ‐Excellent visualization of capitellar OCD via anconeus‐splitting approach ‐Avoids the donor site morbidity of OATS, cost of OCA, and radius of curvature mismatch problems with osteochondral grafting
Limitations
‐Requirement for open elbow arthrotomy ‐Nuanced patient positioning which may require specialized equipment ‐Limited to lesions <6 to 7 mm in depth without deeper bony involvement ‐Limited to contained lesions (intact radial border)
Pearls
‐Careful preoperative planning is crucial to obtain adequate access to capitellar lesion ‐Use pneumatic arm positioner and elbow flexion to gain access to more anterior lesions ‐Meticulous lesion preparation and maintenance of a dry bed field optimizes ECM‐BMAC adherence and incorporation
Pitfalls
‐Avoid use in uncontained lesions as the ECM‐BMAC has no inherent structural integrity at time of initial application ‐Inadequate exposure with too small an incision will limit appropriate graft material placement ‐Early return to activities may compromise healing and long‐term joint health

ECM‐BMAC, extracellular matrix with bone marrow aspirate concentrate; OATS, osteochondral autograft transfer system; OCA, osteochondral allograft; OCD, osteochondritis dissecans.

Regardless of the technique used, preoperative planning is paramount for these cases, and lesion location should dictate the surgical approach. On sagittal MRI sequences, studies have sought to define the location of the lesion by converting the capitellum to a clock face (12 o'clock = proximal, 3 o'clock = anterior). Using this referencing system, the average lesion location is typically between 3 and 5 o'clock but can range from 1:44 to 8:19.^9^, ^10^ The location of these lesions also tends to vary by sport, with more posterior locations in gymnasts and less containment among baseball players.^11^ In a separate article, Johnson et al. evaluated 3 different approaches to the capitellum: posterior anconeus‐split, lateral collateral ligament‐preserving lateral approach, and lateral collateral ligament‐releasing lateral approach.^9^, ^10^ They noted that a posterior anconeus‐split should be used when the lesion is posterior to the 2:46 position, whereas either lateral approach can be used for more anterior lesions.

In conclusion, we describe a method to treat shallow (<6‐7 mm in depth), unstable, but contained capitellar OCD lesions with ECM and BMAC through a mini‐open anconeus‐splitting approach. This approach may be more easily implemented even for those without elbow arthroscopy experience and represents a useful middle‐ground between microfracture and OATS/OCA for capitellar OCD lesions. Although early clinical reports are promising,^1^ future work should continue to assess the long‐term radiologic and functional outcomes of this technique.

## DISCLOSURES

The author (P.D.F.) declares the following financial interests/personal relationships which may be considered as potential competing interests: P.D.F. reports a relationship with *Clinical Orthopedics and Related Research* (CORR) that includes board membership; reports a relationship with BICMD that includes consulting or advisory; reports a relationship with Osso VR that includes consulting or advisory and publishing royalties with Springer Nature. The other authors (J.T.B., N.P‐L., N.H.V., C.Z.) declare that they have no known competing financial interests or personal relationships that could have appeared to influence the work reported in this article.
